# Variability of the Orthopaedic Away Rotation: A Survey of Orthopaedic Program Directors

**DOI:** 10.5435/JAAOSGlobal-D-21-00024

**Published:** 2021-03-10

**Authors:** Travis Blood, Kelly Hill, Symone Brown, Mary K. Mulcahey, Craig P. Eberson

**Affiliations:** From the Department of Orthopaedic Surgery, Harvard Hand and Upper Extremity Fellow, Brigham and Womens Hospital, Boston, MA (Dr. Blood); Department of Orthopaedic Surgery Tulane University School of Medicine, New Orleans, LA (Dr. Hill, Dr. Brown, and Dr. Mulcahey); and The Warren Alpert School of Medicine at Brown University, Rhode Island Hospital/Hasbro Children's Hospital, Providence, RI (Dr. Eberson).

## Abstract

With the increasing interest and pursuit of away rotations by orthopaedic surgery applicants, program directors (PDs) must use information from the 4-week performance to determine who is good fit for their program. For students, despite the increasing cost and time, they are faced with a variable experience from program to program. The purpose of this study was to survey PDs from Accreditation Council of Graduate Medical Education–accredited orthopaedic residency programs to better understand how programs approach the away rotation process.

**Methods:**

An anonymous online survey was distributed to PDs of all 164 accredited allopathic orthopaedic surgery residency programs in the United States. The survey included questions regarding PD demographics, away rotations structure, and the process of interviewing rotating students. The data were aggregated, and an analysis was done.

**Results:**

A total of 61 of 164 (37%) surveys were completed. There was variability regarding the number of away students that a program accepted over the course of a year, and the number of students that a program will accept at one time. Fifty-two of 55 (94%) programs evaluated medical students immediately after their rotation. Visiting students were most commonly evaluated by the program's residents, followed by attendings they rotated with, and only 46% of PDs. Furthermore, PDs placed the most emphasis on work ethic and social interaction when evaluating students compared with surgical skills and orthopaedic knowledge. Only 38.5% of programs reported that visiting students were guaranteed an interview. If granted an interview, 80% of programs require that the students return for interview day.

**Conclusions:**

The visiting rotation has become increasingly more valuable for students; however, there is notable variability in the process between programs. Creating a more standardized away rotation could decrease the variability and facilitate a more beneficial experience to the student and program.

Orthopaedic surgery residency is one of the most competitive categorical subspecialties in the residency match process. According to the 2017 match results from the National Residency Matching Program, there were a total of 1,013 applicants for 727 available orthopaedic residency positions, with 91.9% of matching applicants being from medical schools within the United States.^1^ Students applying for orthopaedic residency are routinely among the top applicants for all specialties regarding United States Medical Licensing Examination (USMLE) Step 1 scores, Alpha Omega Alpha status, and research productivity.^2^ The competitiveness of the orthopaedic match has led applicants to use away rotations as a means of differentiating themselves from other applicants with very similar objective profiles.

Several studies have demonstrated the importance of the orthopaedic away rotation in the match process.^3,4^ Baldwin et al^4^ showed that the number of away rotations, USMLE scores, and more than one orthopaedic home program rotation were the most notable factors influencing a student's ability to match into an orthopaedic residency. The away rotation process benefits both the applicant and the residency program. The away rotation serves as an audition for the applicant at a program of interest, allows the applicant to evaluate the learning and professional environment, and provides an opportunity to obtain a valuable letter of recommendation from a physician other than those at the applicant's home program, while also expanding the student's orthopaedic knowledge base.^5^ Other benefits of the away rotation are for students to get exposure to a variety of residency programs: academic versus community, large versus small programs, and varying didactic educational schedules.

As medical students compete with one another for a limited number of residency positions, residency programs are also competing with one another for the best candidates for their program and can benefit from the away rotation process. Furthermore, the residency program benefits from an expanded 4-week long interview, revealing traits that may be overlooked in a 20- to 30-minute interview or not captured in an application, which may better qualify an applicant for a spot at the program or offer the opportunity for the program to identify an applicant that is a poor fit. Over the course of a rotation, program faculty and residents can better assess the applicant's fit for the program and also evaluate the applicant's surgical skills and knowledge. With the upcoming elimination of Step 1 scores, the away rotation along with research production is likely to become even more important in the process of selecting applicants for residency.^6^

Away rotations or visiting clerkships are generally 2- to 4-week clinical experiences at another institution of interest. Students apply through the Visiting Student Application Service (VSAS), which is a standardized system allowing the submission on an application to multiple programs of interest. Specific programs may ask for supplemental application material, but, in general, the VSAS is the application of choice by most programs.^7^ To apply for an away rotation through VSAS, the student is only required to submit a photograph, curriculum vitae, transcript, and immunization records. Many programs began adding USMLE scores and letters of recommendations as supplemental requirements because the away rotation process has become more competitive.^7^

With the increasing interest and value of away rotations, it is important the students get a valued experience when visiting an institution. To date, little is known about how different programs facilitate and streamline away rotations. The purpose of this study was to survey program directors (PDs) from Accreditation Council of Graduate Medical Education (ACGME) orthopaedic residency programs to better understand how programs organize and structure the away rotation process across the country. This information would provide a framework to create a more standardized away rotation process that would benefit both the candidate and the program.

## Methods

Institutional Review Board approval was obtained from the senior author's institution. The study was designed as a cross-sectional analysis using an anonymous online survey, which was distributed to PDs of 164 accredited allopathic orthopaedic surgery residency programs in the United States using their publicly available contact information. Study data were collected and managed using REDCap electronic data capture tools hosted at the senior author's institution. REDCap (Research Electronic Data Capture) is a secure, web-based application designed to support data capture for research studies, providing (1) an intuitive interface for validated data entry, (2) audit trails for tracking data manipulation and export procedures, (3) automated export procedures for seamless data downloads to common statistical packages, and (4) procedures for importing data from external sources.^8^

The survey consisted of 23 questions regarding visiting rotations at allopathic orthopaedic surgery residency programs (Appendix 1, Supplemental Digital Content 1, http://links.lww.com/JG9/A117). The survey included questions about applicant demographics (eg, number of rotating students), how away rotations are organized and structured at a particular institution, and trends in interviewing visiting students. The survey was constructed by two ACGME residency PDs from two distinct institutions. The survey was not validated before distribution. Follow-up e-mails were sent at 2 and 4 weeks to encourage more participation.

Data were aggregated, and descriptive statistical analysis was done. Responses to individual questions were collected and reported. The focus of the analysis was on the structure of the medical student rotation (eg, number of rotators at one time and acceptance rate of applying visiting students), evaluation of the medical students (who was doing evaluations and presentation required), and how programs interview rotating students. These results were reported as percentages of the responding PDs.

## Results

In total, 61 of 164 (37%) surveys were completed by PDs. The demographics of the PDs are shown in Table 1. Seventy-one percent of the responding PDs held their position for less than 10 years, with 5% of respondents holding their position for 20+ years. Most PDs were associated with a university-based program (82.3%), whereas 14% were associated with a community program and the remaining respondents did not specify. Program size, regarding the number of residents, varied with 65% having between four and seven residents per class and 24% of programs between one and three residents. The length of a given rotation was generally 4 weeks (>90% of programs), with a few programs offering a 2-week rotation.

**Table 1:** Demographics of Program Directors

Sex	Demographics
Male	86.40%
Female	13.56%
Age	Percentage of Respondents
30-39	8.47%
40-49	42.37%
50-59	37.29%
60-69	11.86%
>70	0.00%
Years in practice	
0-5	5.08%
6-10	15.25%
11-15	20.34%
16-20	27.12%
21-25	16.95%
26-30	10.17%
>30	5.08%
Duration of residency director	
0-5	35.59%
6-10	35.59%
11-15	20.34%
16-20	3.39%
>20	5.08%

**Figure 1:**
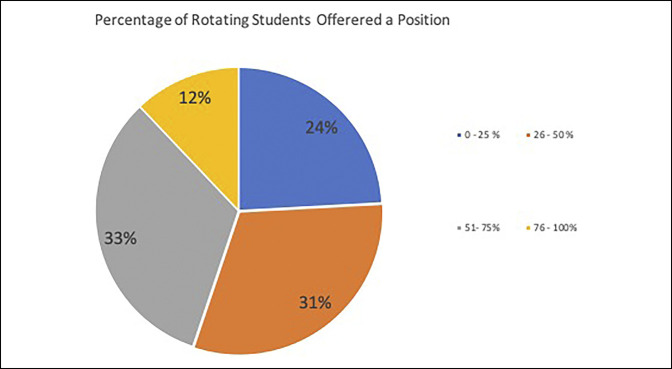
Chart showing acceptance rates reported by program directors of students applying to their program for a visiting clerkship.

**Figure 2:**
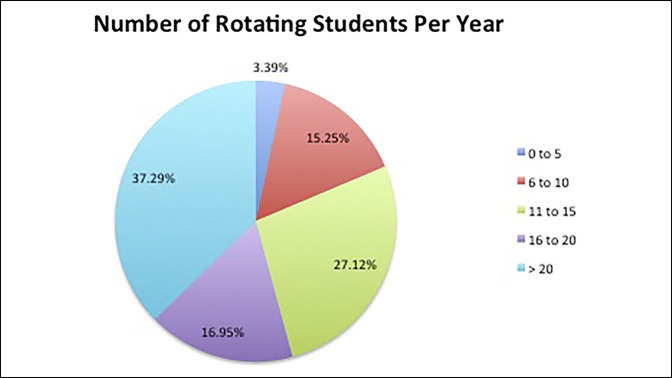
Chart showing variation by percentage of the number of away students at a given time at the responding.

Variability was observed in the percentage of applicants who were offered a position by a program, with 55% of responding programs accepting less than 50% of visiting students' applicants; however, 12% of responding programs accepted all applicants (Figure 1). The number of students that a program will accept during an academic year varied between programs as well, with programs accepting as few as zero to five rotators per year and others accepting more than 20 (Figure 2). Roughly half the programs had between two and four students present during a given rotation block and the other half between four and eight students. There was a small proportion that had greater than eight rotators at a given time.

Regarding the evaluation process, 52 of 55 (94%) programs evaluated medical students after their rotation. Visiting students were most commonly evaluated by the program's residents (39/50, 78%) and attending surgeons (38/50, 76%), whereas only 23 of 50 (46%) PDs evaluated rotating students. Programs used feedback from residents (36/50, 72%), a one-on-one meeting with the attending on service (25/50, 50%), and 28 of 50 (56%) programs used a formal grading process. Furthermore, PDs stated that they place the most emphasis on work ethic (22/51, 43%) and social interaction (20/51, 39%) when evaluating students during their away rotations compared with surgical skills (zero) and orthopaedic knowledge (4/51, 8%).

Although most programs (37/54, 68.5%) require a visiting student to give a presentation as an evaluation process, there was variability in the length (range, 5 to 30 minutes) and audience among the institutions.

Furthermore, only 20 of 52 (38.5%) PDs reported that a visiting student is guaranteed an interview. If granted an interview, 41 of 52 (80%) programs require that the students return on the program's interview day. Of the programs that do not guarantee an interview, 20 of 32 (62.5%) give >75% of visiting students an interview.

## Discussion

Substantial heterogeneity exists among orthopaedic surgery residency PDs for the organizational structure of medical student away rotations. The response rate from PDs was lower than expected at 38%. This may be attributed to the busy schedules of PDs trying to balance a clinical practice and oversee a residency program in which they do not have time to complete the study. It may also reflect the interest of the PDs toward the away rotation process. Most responses came from young PDs, which may indicate that they are more actively involved and engaged in the residency program and away rotation process. Although less than we would have liked, we think that the responses are enough to get a glimpse of how programs run the visiting student rotation.

Programs accepted anywhere from zero to two students to over 20 students per academic year, and between two and eight students at a given time. Some programs accepted only 55% of away rotations applicants and other programs accepting all away rotations applicants. This variability in acceptance rates from program to program may be influenced by program size and ability to accommodate student rotators. Larger programs likely have more access and ability to accommodate visiting students by having more surgeons available for mentorship. Similarly, the more competitive programs are likely able to be more selective of visiting students and therefore may have a lower acceptance rates and take fewer applicants. With the increasing number of students doing away rotations, these discrepancies between programs are only likely to grow.

Previous studies have demonstrated that PDs value away rotation performance more than the residency application alone.^4,9^ Although this may be true, we found that there is little consensus in the overall evaluation process for visiting students. Most PDs reported that their program evaluated students after their rotation; however, only approximately half of the programs had a formal evaluation process. Most programs relied on the interactions of the students with residents and attendings for evaluation. Without a formal process, most programs rely on subjective rather than objective information to evaluate the rotating students. We think that a more structured evaluation process would help standardize the away rotation process. A formal evaluation process would be similar to standardizing the applicant selection process for residency, which has been shown to be reliable and predicable.^10^ Particularly in the era of medical schools moving toward pass/fail grading for the first 2 years, elimination of a USMLE Step 1 score, absence of class rank information availability from an increasing number of medical schools, variability in the grading systems used in the third year clinical rotations, and evaluating an applicant's basic fund of knowledge, work ethic, and personality traits may be best done first-hand through the away rotation process.^11^

With applicants spending a notable portion of their fourth year on home and away orthopaedic rotations, structuring a rotation to maximize educational content for the student is crucial. Furthermore, PDs value work ethic and social interaction more than orthopaedic knowledge and surgical skills when evaluating visiting students. Surgical training and musculoskeletal education are often underrepresented in medical schools, and many students lack the skills to do simple procedures or basic orthopaedic knowledge; therefore, evaluating visiting students' surgical ability is difficult in the assessment of their potential to become a surgeon.^12^ However, having students do simple tasks such as suturing or placing screws in a plate during fracture fixation, as well as incorporating simulation and virtual reality platforms, may facilitate the assessment of students' ability to become a skilled surgeon. Future studies should investigate the relationship between medical student surgical skill and skill level after training.

Completing an away rotation at a particular institution has been shown to increase a residency applicant's odds of obtaining an interview by 1.5 times.^13^ In our study, 38.5% of programs guaranteed visiting students an interview. Winterton et al^14^ surveyed fourth-year medical students regarding the application process, and the authors found that the average fourth year applied to 65 residency programs with a goal of 15 interview offers, or 23.1%. Furthermore, of the programs that offer an interview to a visiting student, 80% of them require the student to return on the interview day. The other 20% of programs have an exit interview at the end of the visiting rotation. The average visiting student spends $958 dollars per away rotation with the average applicant completing 2.1 away rotations.^13^ Having to return for an interview adds considerable cost to an already expensive away rotation. Therefore, PDs may consider interviewing visiting students at the conclusion of their away rotation to help decrease the cost of the application/interview process.

Medical schools seem to be moving toward pass/fail grading and a reluctance to differentiate the students through class ranking. Orthopaedic residency programs that use USMLE Step 1 scores to screen residency applications will no longer be able to do so; therefore, the away rotation is likely to assume an even greater importance in selecting residents. Bearing that in mind, it is important that away rotations be structured in a manner that allows for thorough evaluation of the student while also providing a sound educational experience.

## Conclusion

Our analysis shows that there is considerable variability in how orthopaedic away rotations function from program to program. Not all programs need to be the same, but we think that PDs and the ACGME need to be aware of the variations that exist. Creating a more standardized away rotation process and experience may decrease the variability and facilitate a more beneficial experience for medical students and orthopaedic residency programs.
